# Phenotypic Analysis of the Anti-*T. cruzi* Activity of Natural Products Obtained from Brazilian Botanical Sources

**DOI:** 10.3390/molecules31162790

**Published:** 2026-08-11

**Authors:** Ludmila Ferreira de Almeida Fiuza, Denise da Gama Jaen Batista, Carolinna Silva Bressan, Marcos Meuser Batista, Raquel Silva de Azevedo, Ketlym da Conceição, Vagner Pereira da Silva, Ana Luíza Rangel Bérenger, Flávia da Cunha Camillo, André Mesquita Marques, Maria Raquel Figueiredo, Maria de Nazaré Correia Soeiro

**Affiliations:** 1 Laboratório de Biologia Celular, Instituto Oswaldo Cruz (IOC), Fundação Oswaldo Cruz (FIOCRUZ), Rio de Janeiro 21040-900, Rio de Janeiro, Brazil; ludmila.fiuza@ioc.fiocruz.br (L.F.d.A.F.); denise021278@hotmail.com (D.d.G.J.B.); carolinna.bressan@ioc.fiocruz.br (C.S.B.); meusermb@ioc.fiocruz.br (M.M.B.); raquel.azevedo@ioc.fiocruz.br (R.S.d.A.); ketlymconceicao@ioc.fiocruz.br (K.d.C.); 2 Departamento de Produtos Naturais (TecBio-LDFito), Instituto de Tecnologia em Fármacos (Farmanguinhos), Fundação Oswaldo Cruz (FIOCRUZ), Rio de Janeiro 21041-250, Rio de Janeiro, Brazil; vagner.silva@fiocruz.br (V.P.d.S.); ana.berenger@fiocruz.br (A.L.R.B.); flavia.camillo@fiocruz.br (F.d.C.C.); andre.marques@inpp.gov.br (A.M.M.); maria.figueiredo@fiocruz.br (M.R.F.); 3 Instituto Nacional de Pesquisa do Pantanal (INPP), Cuiabá 78060-900, Mato Grosso, Brazil

**Keywords:** *Trypanosoma cruzi*, Chagas disease, Piperaceae, natural products, drug screening

## Abstract

Chagas disease, caused by *Trypanosoma cruzi* (*T. cruzi*), affects more than 6 million people worldwide, predominantly in Latin America. Current chemotherapy relies on Benznidazole (Bz) and Nifurtimox, which are associated with adverse effects and limited efficacy during chronic infection, underscoring the need for new therapeutic options. Natural products remain an important source of bioactive molecules for antiparasitic drug discovery. In this study, 34 extracts obtained from 18 Brazilian plant species were evaluated for anti-*T. cruzi* activity. After cytotoxicity assessment, extracts were screened against intracellular amastigotes (Tulahuen strain, DTU VI) and bloodstream trypomastigotes (Y strain, DTU II). Most samples exhibited low toxicity toward mammalian cells (LC_50_ > 120 μg/mL). Among them, the inflorescence extract of *Piper claussenianum* (Miq.) C. DC. (PCFLD) showed the highest activity. At 20 μg/mL, PCFLD reduced intracellular parasite burden by 94%, with an EC_50_ of 3.6 ± 0.36 μg/mL and a selectivity index of 20. Against trypomastigotes, the extract displayed an EC_50_ of 11.56 ± 5.88 μg/mL and a selectivity index of 11. In an acute murine model, PCFLD (10 mg/kg/day) reduced peak parasitemia by 40%, outperforming Bz at the same dose. These findings identify *P. claussenianum* as a promising source of anti-*T. cruzi* compounds.

## 1. Introduction

Neglected tropical diseases (NTDs) pose a major global public health challenge, particularly in low- and middle-income regions, where socioeconomic and environmental factors contribute to their persistence and transmission [1,2]. Among these diseases, Chagas disease (CD) is of particular importance. Present in the Americas for more than 9000 years, CD, also known as American trypanosomiasis, was first described by Carlos Chagas in 1909 [3,4]. Previously restricted to rural areas of Latin America, CD has spread beyond endemic regions due to increased migration and population mobility, which have overcome geographical barriers. As a result, cases have been reported in non-endemic regions, including North America, Europe, and Asia [5,6,7].

This silent and progressive infection is caused by *Trypanosoma cruzi* (*T. cruzi*). This protozoan exhibits two proliferative forms—epimastigotes (in the insect vector) and amastigotes (in mammals, including humans)—and one non-replicative infective form, the trypomastigote, which is present in both the vector and the vertebrate host [8,9]. Transmission occurs through different mechanisms, including the classical vector-borne route mediated by triatomine insects of the family Reduviidae, commonly referred to as “kissing bugs” [10,11]. In addition to the classical vector-borne route, *T. cruzi* can be transmitted orally through the ingestion of food contaminated with triatomine feces or infected insects. Other transmission routes include congenital transmission, blood transfusion, organ transplantation, and laboratory accidents, among others of lesser epidemiological relevance [12,13,14,15,16].

From a clinical perspective, CD presents distinct phases characterized by different clinical and parasitological features. The acute phase, which lasts up to approximately eight weeks after infection, is generally asymptomatic or oligosymptomatic, although some patients may present nonspecific manifestations [17,18]. During this phase, patent parasitemia is observed and can be detected by conventional diagnostic methods, including light microscopy. Due to the activation of the host immune response, parasite replication is partially controlled; however, complete elimination of the infection is rarely achieved, allowing the persistence of the parasite and progression to the chronic phase [17,19,20]. During the chronic phase, most infected individuals remain in the indeterminate form, characterized by the absence of apparent clinical manifestations despite the presence of persistent infection. However, approximately 30–40% of patients may develop clinical complications years or even decades after the initial infection. Chronic Chagas cardiomyopathy (CCC) represents the most frequent and severe clinical outcome, being a major cause of morbidity and mortality among patients with Chagas disease [20,21,22].

Despite its epidemiological relevance, therapeutic options for Chagas disease remain limited, with fewer than 1% of infected individuals having access to etiological treatment. Current therapy relies on benznidazole (Bz) and nifurtimox (Nf) [23,24,25]. Both drugs are effective in the acute phase but less effective in chronic Chagas disease. These limitations highlight the need for safer therapies, including drug repurposing and combination strategies [26,27,28,29].

In this context, natural products are a valuable source of new drug candidates for neglected tropical diseases due to their structural diversity and broad biological activity [30,31].

Natural products have long played a key role in drug discovery. Quinine, isolated from *Cinchona officinalis* (Rubiaceae family), was the first effective antimalarial drug and inspired the development of synthetic derivatives [32,33]. Similarly, flavonoids and saponins have shown promising activity against *Leishmania donovani*, reinforcing the potential of natural products as sources of new therapies [34].

In the context of CD, natural products have also emerged as promising sources of new anti-*T. cruzi* candidates. Studies from our group [35] investigated through in silico and in vitro approaches and demonstrated that *Plumbago auriculata* Lam root extract and its major constituent, plumbagin, exhibited potent activity against intracellular amastigotes and bloodstream trypomastigotes, with efficacy comparable or superior to Bz, highlighting the potential of botanical-derived compounds for Chagas disease drug discovery.

Considering the promising trypanocidal activity of natural products, this study aimed to perform a phenotypic screening of extracts obtained from Brazilian botanical sources. A total of 18 plant species belonging to different botanical families—including Anacardiaceae, Asteraceae, Chrysobalanaceae, Clusiaceae, Fabaceae, Lauraceae, Lecythidaceae, Linaceae, Marcgraviaceae, Myrtaceae, Piperaceae, Sapotaceae and Solanaceae—were investigated to identify natural products with anti-*T. cruzi* activity and evaluate their potential as candidates for the development of new therapeutic alternatives for Chagas disease.

## 2. Results

### 2.1. Toxicity Profile Analysis

The cytotoxicity of the extracts was initially evaluated using uninfected L929 fibroblast cultures. Cells were exposed to increasing concentrations of each extract for 96 h under standard culture conditions. After the incubation period, cellular morphology was examined by light microscopy, and cell viability was determined using the AlamarBlue^®^ (resazurin) colorimetric assay. These analyses allowed the characterization of the cytotoxic profile of each extract and the identification of concentrations suitable for subsequent antiparasitic screening.

Regarding cytotoxicity, after 96 h of exposure to concentrations ranging from 0 to 120 μg/mL, only the extracts PCFLD (*Piper claussenianum* Miq. C. DC., Piperaceae family, inflorescences) and STFE70PAc (*Schinus terebinthifolius* Raddi, Anacardiaceae family, leaf) exhibited a low cytotoxic profile toward L929 fibroblasts. These extracts showed LC_50_ values ranging from 72 to 79 μg/mL—Table 1. In contrast, all other extracts, as well as the reference drug, did not induce detectable toxic effects up to the highest concentration tested (120 μg/mL) Table 1. Light microscopic examination confirmed the preservation of cellular morphology and monolayer integrity for the treated samples.

### 2.2. Extracts Trypanocidal Activity Evaluation

Following the cytotoxicity assessment, the antiparasitic activity of the 34 natural products was evaluated in vitro according to the drug-screening methodology as established by Romanha et al. [36] and Soeiro et al. [29]. In an initial screening step, all samples were tested at a fixed concentration to identify the most promising candidates against the intracellular amastigote form of *T. cruzi* (Tulahuen-β gal strain—DTU VI), after 96 h of incubation. Among the 34 evaluated compounds, 33 extracts exhibited no antiparasitic activity, resulting in parasite reduction rates up to 22% compared with untreated controls while Bz gave 89% of parasitism decline. Under this protocol, the extract PCFLD demonstrated a remarkable antiparasitic effect, with activity comparable to the reference drug. PCFLD induced a 94% reduction in the intracellular parasite burden—Table 2.

The pronounced activity observed for PCFLD, together with its cytotoxic profile previously determined in L929 fibroblasts, highlights this extract as a promising candidate for further pharmacological evaluation. Therefore, PCFLD was selected for subsequent dose–response assays to determine its half-maximal effective concentration (EC_50_) and to better characterize its antiparasitic potency and selectivity. The results showed that the extract PCFLD, obtained from the inflorescences of *P. claussenianum*, exhibited antiparasitic activity against *T. cruzi* amastigotes, with an EC_50_ value of 3.6 ± 0.36 μg/mL. In comparison, the reference drug showed an EC_50_ value of 0.17 ± 0.007 μg/mL (Table 3).

The cytotoxicity profile allowed the determination of the selectivity index (SI = ratio between the LC_50_ (mammalian cells toxicity) and the EC_50_ (antiparasitic activity) values). PCFLD displayed a SI = 20, whereas Bz is >700. Despite being less potent than the reference drug, the extract demonstrated a favorable antiparasitic activity combined with an acceptable selectivity profile.

Next, we evaluated the activity of the extracts against the infective stage of *T. cruzi* relevant to mammalians, the bloodstream trypomastigote form (BT). To this end, non-replicative trypomastigotes were incubated for 24 h with increasing concentrations of the crude extracts and the reference drug Bz, followed by assessment of parasite viability by light microscopy.

Among the 34 extracts tested, six samples (STFE70, STFE70PAc, SBFE, LNFE70, PCFH, and PCFLD) displayed trypanocidal activity against *T. cruzi* trypomastigotes, with EC_50_ values ranging from 11 to 33 μg/mL (Table 3).

Among these extracts, PCFLD, obtained from *P. claussenianum* (Piperaceae), was again particularly noteworthy. This dichloromethane extract had previously shown the highest activity against intracellular amastigotes (Table 3) and demonstrated the strongest trypanocidal effect against bloodstream trypomastigotes compared to the other samples, displaying an EC_50_ value of 11.56 ± 5.88 μg/mL. Under the same experimental conditions, Bz exhibited an EC_50_ value of 3.8 μg/mL (Table 3).

To inspect potential cardiotoxicity, viability assays using H9c2(2-1) rat cardiomyoblast cells were performed. The results demonstrated that neither the extracts nor the reference drug induced detectable cardio toxic effects at the highest concentrations tested (120 μg/mL). Based on the cytotoxicity and antiparasitic activity against BT, the PCFLD extract presented a selectivity index of greater 11—Table 3.

Taken together, these findings highlight the antiparasitic potential of the PCFLD extract obtained from *P. claussenianum* inflorescences. Among all extracts evaluated, PCFLD consistently exhibited the highest activity against both intracellular amastigotes (Tulahuen strain) and bloodstream trypomastigotes (Y strain) of *T. cruzi*, while maintaining a favorable selectivity profile toward mammalian cells. Owing to its superior potency and selectivity compared with the other extracts tested, PCFLD emerged as the most promising candidate and was therefore selected for further evaluation of its in vivo efficacy in a mouse model of acute *T. cruzi* infection.

To evaluate the in vivo antitrypanosomal activity of the most active extract (PCFLD), a proof-of-concept study was performed using an acute murine model of *T. cruzi* infection. The effects of treatment on parasitemia progression and survival were assessed and compared with those obtained using Bz.

In the acute murine model of *T. cruzi* infection, PCFLD administered at 10 mg/kg/day (i.p.) did not trigger detectable toxic events until the end of the treatment. Since the 10 mg/kg/day dose showed no evidence of toxicity, it was selected for the evaluation of antiparasitic activity in a murine model, while Bz, used as the reference drug, was administered at both a subtherapeutic dose of 10 mg/kg/day and its optimal therapeutic dose of 100 mg/kg/day for comparison.

**Figure 1:**
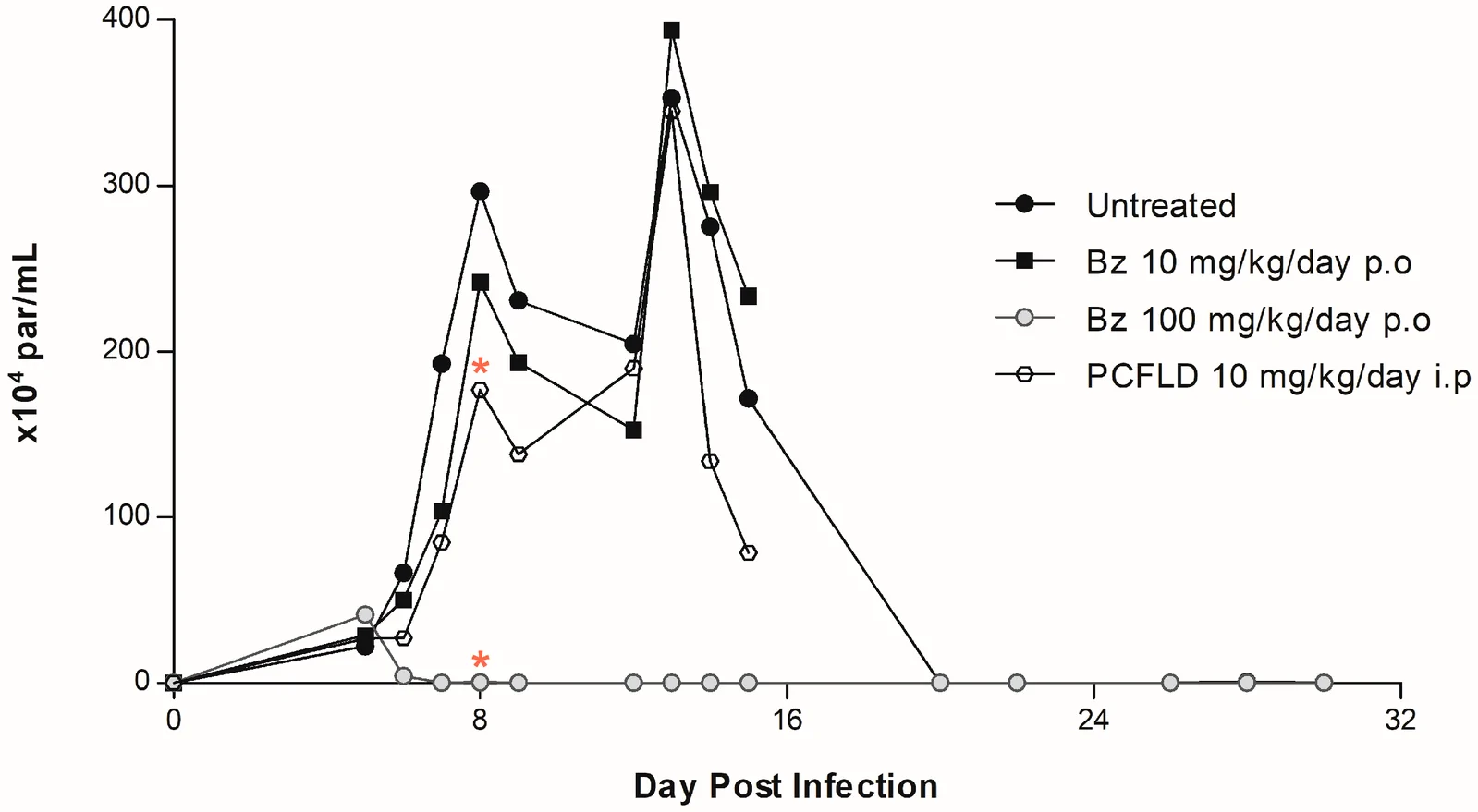
The effects of dichloromethane extract of *P. claussenianum* (PCFLD) and benznidazole (Bz) on parasitemia curve in *T. cruzi*-infected male Swiss mice. The infected animals (*n* = 5 per group) were treated or not for 5 consecutive days using PCFLD (10 mg/kg/day) and Bz (10 and 100 mg/kg/day), starting the drug administration at the parasitemia onset (5 dpi). *p* < 0.05 versus the untreated control group is indicated by a red asterisk (*).

Regarding antiparasitic activity, PCFLD reduced peak parasitemia by 40%, representing a significant reduction (*p* = 0.0039) compared with the untreated group and demonstrating relevant in vivo antitrypanosomal activity. At the same dose, Bz at a suboptimal dose (10 mg/kg/day) reduced parasite burden by only 18%, whereas the reference drug administered at its optimal dose (100 mg/kg/day) completely suppressed parasitemia, corresponding to a significant 100% reduction (*p* < 0.0001)—Figure 1. Thus, PCFLD was approximately two-fold more effective than Bz when administered at the equivalent dose of 10 mg/kg/day, highlighting its potential as a promising source of bioactive compounds for the development of new therapeutic strategies against Chagas disease. Regarding mortality, untreated animals, as well as those treated with Bz at 10 mg/kg and the extract at 10 mg/kg, began to die at 12 dpi. In contrast, no mortality was observed in animals treated with the reference drug at its optimal dose (100 mg/kg).

## 3. Discussion

Chagas disease is classified as a neglected tropical disease (NTD) that disproportionately affects socially and economically vulnerable populations. Despite its substantial public health and socioeconomic burden, millions of infected individuals worldwide remain undiagnosed or lack adequate access to treatment. Moreover, investments in research and drug development remain insufficient relative to the magnitude of the disease [37,38,39]. The discovery and development of new drugs is a lengthy, complex, and costly process, often requiring more than a decade before clinical approval. As a result, diseases that predominantly affect low-income populations receive limited attention from the pharmaceutical industry, leading to a restricted pipeline of therapeutic candidates [29].

Currently, the etiological treatment of Chagas disease relies mainly on benznidazole (Bz) and nifurtimox (Nf). Although both drugs are effective during the acute phase of infection, their use is limited by frequent adverse effects, prolonged treatment regimens, and reduced efficacy in chronically infected patients. In addition, no vaccine is currently available, underscoring the need for safer and more effective therapeutic strategies capable of targeting multiple stages of the parasite life cycle [40,41]. In this context, several strategies have been employed to identify new drug candidates. These include phenotypic screening of synthetic compound libraries and natural products, drug repurposing, the evaluation of combination therapies, and the rational design of molecules targeting biochemical pathways essential for parasite survival and virulence.

Natural products deserve particular attention, as their remarkable chemical diversity and structural complexity constitute a valuable source of novel molecular scaffolds with the potential to generate innovative antiparasitic agents and contribute to the development of more effective therapies for Chagas disease [31,42,43]. Considering the evidence of the antitrypanosomal activity of natural products [44], this study aimed to evaluate botanical extracts from different Brazilian plant species to identify promising candidates for the treatment of Chagas disease. In total, 34 extracts obtained from different botanical families (Anacardiaceae, Asteraceae, Chrysobalanaceae, Clusiaceae, Fabaceae, Lauraceae, Lecythidaceae, Linaceae, Marcgraviaceae, Myrtaceae, Piperaceae, Sapotaceae, and Solanaceae) were assessed using well-established screening protocols [29].

Cytotoxicity evaluation showed that most extracts presented a favorable safety profile in L929 fibroblasts, with no detectable toxic effects at concentrations up to 120 μg/mL. Only PCFLD (*P. claussenianum*, Piperaceae) and STFE70PAc (*S. terebinthifolius*, Anacardiaceae) exhibited low cytotoxicity, with LC_50_ values ranging from 72 to 79 μg/mL, supporting their further evaluation in antiparasitic assays.

Following the initial screening against the intracellular amastigote form of *T. cruzi*, most samples exhibited limited antiparasitic activity, with parasite reduction rates below 22% under the evaluated conditions. In contrast, PCFLD exhibited remarkable activity, inducing a 94% reduction in the intracellular parasite burden, comparable to that observed for the reference drug (89%).

The low cytotoxicity (in vitro and in vivo) and remarkable antiparasitic activity observed for PCFLD from *P. claussenianum* against the intracellular amastigote form of *T. cruzi* are consistent with previous reports describing the promising effect of *Piper* species against protozoan parasites. In particular, Marques et al. [45] reported that essential oils obtained from the leaves and inflorescences of *P. claussenianum* exhibited leishmanicidal activity against different forms of *L. amazonensis*. These oils presented a rich composition of monoterpenes and sesquiterpenes, with sesquiterpenes predominating in leaf samples and monoterpenes being more abundant in inflorescence oils. Both essential oils inhibited parasite growth, with the leaf-derived oil showing higher activity, presenting an EC_50_ value of approximately 30.4 μg/mL against *L. amazonensis*. Furthermore, treatment of infected macrophages with the leaf oil resulted in a 95% reduction in infection rates, further supporting the antiparasitic potential of *P. claussenianum* metabolites.

Similarly, essential oil from *Piper callosum* demonstrated significant activity against both the amastigote and promastigote forms of *L. amazonensis*, while exhibiting low cytotoxicity toward VERO cells and a favorable selectivity index [46]. These findings are also in agreement with previous results obtained by our research group for another member of the Piperaceae family. Peres et al. [47] reported that the crude extract of *Piper tectoniifolium* exhibited significant activity against the intracellular forms of *T. cruzi*, with an EC_50_ value of 12.85 ± 1.52 μg/mL, while maintaining a favorable cytotoxic profile toward L929 fibroblasts.

Considering the relevance of bloodstream trypomastigotes in *T. cruzi* infection and dissemination, the promising activity of PCFLD from *P. claussenianum* was further evaluated against this infective form. PCFLD exhibited the strongest trypanocidal effect among the tested extracts, with an EC_50_ value of 11.56 ± 5.88 μg/mL against bloodstream trypomastigotes, in addition to its potent activity against intracellular amastigotes (EC_50_ = 3.6 ± 0.36 μg/mL). Importantly, PCFLD also showed a favorable safety profile, with no detectable cytotoxic effects toward H9c2 cells at concentrations up to 120 μg/mL. These findings are consistent with previous results obtained by our research group for another Piperaceae species. Peres et al. [47] demonstrated that the crude extract of *Piper tectoniifolium* (PTFrE) exhibited an EC_50_ value of 38.8 ± 2.1 μg/mL against bloodstream trypomastigotes, while displaying low cardiotoxicity in cell cultures, with an LC_50_ value of 124.2 ± 6.8 μg/mL.

According to the current literature, for natural products or synthetic compounds, it is recommended to use a fixed concentration in the first stage of a phenotypic screening analysis [29]. Only those compounds displaying rather similar or better activity than Bz undergo a concentration–response curve to define their respective EC_50_ values as we presently performed. Also, following the current consensus, an active or “hit” substance/compound must ideally reach selectivity indexes (SI) > 10, and PCFLD gave SI = 20 against intracellular forms, confirming its promising trypanosomicidal effect in vitro. Collectively, the antiparasitic activity and favorable cytotoxicity profile observed for PCFLD supported its selection for further evaluation in vivo.

Then, this subsequent analysis in an experimental mouse model demonstrated that PCFLD has antitrypanosomal activity, reducing peak parasitemia by 40% and outperforming Bz at the same dose. Although its efficacy was lower than that achieved by the reference drug at the standard therapeutic dose (100 mg/kg/day), the observed reduction in parasite burden highlights the potential of PCFLD as a source of bioactive compounds for Chagas disease drug discovery. These findings are consistent with previous reports describing the antitrypanosomal activity of Piperaceae-derived metabolites. García-Huertas et al. [48] demonstrated that a lignan isolated from *Piper cubeba* significantly reduced bloodstream trypomastigote levels in infected mice, with the greatest effect observed at 30 mg/kg.

PCFLD is derived from *P. claussenianum*, a species belonging to the Piperaceae family and the genus *Piper*. This genus stands out as an important source of bioactive secondary metabolites, with numerous species exhibiting well-documented medicinal and pharmacological properties. In Brazil, *Piper* species represent a significant proportion of the approximately 500 Piperaceae species recorded in the country [45,49]. Previous phytochemical investigations demonstrated that *Piper* species are rich sources of structurally diverse secondary metabolites such as flavonoids monoterpenes, sesquiterpenes, arylpropanoids, methylenedioxyphenyl derivatives, alkaloids, lignans, and amides, many of which exhibiting antimicrobial, antioxidant, anti-inflammatory, and antiparasitic activities [50].

The HPLC chromatographic analysis provides a valuable chemical fingerprint of the extracts [50]. The present HPLC of the most active extract (PCFLD), with detection at 254 and 365 nm, revealed the predominance of compounds eluting in the chromatographic region characteristic of flavonoids. The dichloromethane extract was fractionated and purified by silica gel column chromatography, allowing the isolation of 5-methoxy-7-hydroxydihydroflavone, 5,7-dihydroxydihydroflavone, and 2′,6′-dihydroxy-4′-methoxychalcone. The structures of the isolated compounds were elucidated by 1H and 13C nuclear magnetic resonance (NMR) spectroscopy as depicted in Figures S1 and S2.

Taken together, these findings demonstrate that the inflorescence extract of *P. claussenianum* stood out due to its activity against intracellular amastigotes and bloodstream trypomastigotes, combined with an acceptable cytotoxicity profile, supporting its classification as a potential Hit candidate [51]. The reduction in parasitemia observed in infected mice further reinforces the anti-*T. cruzi* potential of this extract and highlights the relevance of natural products, particularly those from the Piperaceae family, as sources of bioactive compounds. These efforts will contribute to understanding the mechanisms underlying its antitrypanosomal activity and may support the development of new therapeutic strategies for the treatment of Chagas disease.

## 4. Materials and Methods

### 4.1. Plant Material

Fruits, seeds, bark, stems, branches, leaves and inflorescences of the plant species were collected in the states of Minas Gerais, Rio de Janeiro, and Espírito Santo—Brazil (Table S1). Representative voucher specimens of each species were deposited in Herbariums located in Rio de Janeiro, Brazil. The plant material was registered in the National System for the Management of Genetic Heritage and Associated Traditional Knowledge (SisGen) under registration number AB5D582 by Dr. Maria Raquel Figueiredo (FarManguinhos/Fiocruz), in accordance with the provisions of Law No. 13.123/2015 and its current regulations.

### 4.2. Plant Extraction

Fruits, seeds, bark, stems, branches, leaves and inflorescences were collected, separated, dried at 40 °C and reduced to small fragments, approximately 5.0 mm in diameter. Each plant part was individually subjected to dynamic maceration using different organic solvents: ethanol (the preferred solvent), ethyl acetate, methanol, dichloromethane, and hexane. Extractions were performed separately with each solvent using a plant material-to-solvent ratio of 1:10 (*w*/*v*) for 72 h at room temperature. After the extraction process, the extracts were filtered and the respective solvents were removed under reduced pressure, resulting in crude extracts—specific to each plant part and solvent employed. Thus, crude extracts were obtained from the different plant organs using distinct solvents, as shown in Table 4. The SBFE and STFE70 extracts were fractionated by liquid–liquid partitioning using ethyl acetate, yielding the SBFEPAc and STFE70PAc fractions, respectively. The extracts were then properly stored until subsequent biological evaluation. All extraction procedures were carried out at the Natural Products Laboratory (TecBio/LDFito), Farmanguinhos/FIOCRUZ.

### 4.3. Compounds

Bz was used as the reference drug in all assays. For the in vitro assays, Bz was obtained from Farmanguinhos/Fiocruz, donated by Dr Nubia Boechat, whereas for the in vivo experiments, the reference drug was purchased from Laboratório Farmacêutico do Estado de Pernambuco (Recife, Brazil). Samples were prepared as stock solutions in dimethyl sulfoxide (DMSO; Sigma-Aldrich, St. Louis, MO, USA) and diluted to the working concentrations while ensuring that the final DMSO concentration did not exceed levels associated with toxic effects on mammalian or parasitic cells (0.6% in the in vitro assays and 10% in the in vivo experiments) [47].

### 4.4. Mammalian Cells

The L929 cell cultures were maintained under routine laboratory conditions through weekly passages using 0.01% trypsin solution, followed by cell seeding in 96-well plates at a density of 4 × 10^3^ cells per well, according to the protocol previously described by Romanha et al. [36]. Briefly, cells were regularly monitored for confluence and morphological characteristics to ensure adequate growth conditions before experimental procedures.

Cardiac cells from the H9c2(2-1) cell line were maintained through successive passages in supplemented Dulbecco’s Modified Eagle Medium (DMEM—Sigma-Aldrich, St. Louis, MO, USA) at 37 °C under a humidified atmosphere containing 5% CO_2_. The culture medium was routinely replaced, and cell growth was monitored until an appropriate confluence level was reached, following previously established conditions [52]. The cells were subsequently used for cytotoxicity and biological activity assays.

### 4.5. Citotoxicity Assays

H9c2(2-1) and L929 cells were exposed to increasing concentrations of the compounds, which were serially diluted in Eagle’s medium or RPMI-1640 medium (Sigma-Aldrich, St. Louis, MO, USA) supplemented with 1% L-glutamine and incubated for 24 and 96 h, respectively [53]. After treatment (0, 15, 30, 60 and 120 µg/mL) of the extracts as well as the reference drug, cellular morphology was evaluated by light microscopy, considering parameters such as cell density, contractility, and cytoplasmic vacuolization. Cell viability was further determined using PrestoBlue (Invitrogen, Thermo Fisher Scientific, Waltham, MA, USA) for the cardiac cells and AlamarBlue (Invitrogen, Thermo Fisher Scientific, Waltham, MA, USA) for the L929 assay, based on fluorescence and absorbance measurements (560–590 nm and 570–600 nm, respectively) using a spectrophotometer (Molecular Devices, San Jose, CA, USA) [47,53]. The percentage of viable cells was calculated, and the cytotoxicity profile was expressed as LC_50_ values, corresponding to the concentration of each compound capable of reducing cell viability by 50%.

### 4.6. Parasites

Trypomastigote and intracellular forms of the Tulahuen-β-gal strain (DTU VI) were obtained using L929 cell cultures at a host-to-parasite ratio of 1:10 [36]. Cell-derived trypomastigotes from the Tulahuen β-gal strain were collected from the supernatant of previously infected L929 cell cultures maintained at 37 °C under a humidified atmosphere containing 5% CO_2_. After 48 h of host–parasite interaction, the cultures were washed with phosphate-buffered saline (PBS, pH 7.4). After six days of infection, the trypomastigotes released into the culture supernatant were collected. The supernatant was then centrifuged at 4200 rpm for 10 min to recover the parasites in the cell pellet [53]. For purification of Bloodstream trypomastigotes (Y strain), Swiss male mice were infected with 10^5^/mice via ip. At the parasitemia peak, the blood was collected by heart punction and parasite purified as reported [35].

### 4.7. Trypanocidal Activity Assay

To evaluate the anti-*T. cruzi* effect upon intracellular forms L929 cells (4 × 10^3^ cells/well) were seeded into sterile 96-well microtiter plates and infected for 2 h with trypomastigotes of the Tulahuen-β-gal strain (4 × 10^4^ parasites/well) at a host cell-to-parasite ratio of 1:10. After 48 h of infection establishment, fractions were added (initially, the samples were evaluated at fixed concentration of up to 20 µg/mL, followed by exposure to increasing concentrations ranging from 0 to 20 µg/mL) and the infected cultures were maintained at 37 °C for an additional 96 h. Parasite burden was assessed by adding the substrate chlorophenol red-β-D-galactopyranoside (CPRG), followed by spectrophotometric measurement at 570 nm. Benznidazole was used as the reference drug [52,53].

Bloodstream trypomastigotes of the Y strain (5 × 10^6^ parasites/mL) were incubated at 37 °C in RPMI medium in the presence or absence of serial dilutions of the fractions and the reference drug (0–40 µg/mL). After 24 h of incubation, parasite viability was evaluated by light microscopy through the direct quantification of viable parasites using a Neubauer chamber. Parasite morphology and motility were also monitored during the analysis to determine the effects induced by the treatments. The effective concentration required to reduce the parasite population by 50% (EC_50_) was subsequently calculated based on the percentage of parasite survival obtained for each tested sample [54].

### 4.8. In Vivo Assay

Male Swiss Webster mice (18–20 g; 4–5 weeks old) were obtained from the Institute of Science and Technology in Biomodels (ICTB-FIOCRUZ). The animals were housed in groups of a maximum of five mice per cage and maintained in a specific pathogen-free (SPF) facility under controlled environmental conditions, with a temperature range of 20–24 °C and a 12 h light/12 h dark cycle. Mice received sterilized water and standard laboratory food ad libitum throughout the experimental period. Before the beginning of the experiments, the animals were allowed to acclimatize for seven days under the same housing conditions [55].

For a proof-of-concept study, animals were infected by intraperitoneal (i.p.) administration of 10^5^ bloodstream trypomastigotes (Y strain), and treatment was initiated at the onset of parasitemia (5 days post infection—dpi), including only mice with detectable parasitemia. Age-matched control mice were housed under identical conditions. The following experimental groups were used (5 mice per group): untreated (infected vehicle-treated control) and treated (infected and treated with PCFLD and Bz). The PCFLD extract was administered intraperitoneally once daily for five consecutive days at a dose of 10 mg/kg, whereas Bz was administered orally by gavage once daily for the same period at doses of 10 and 100 mg/kg.

**Figure 2:**
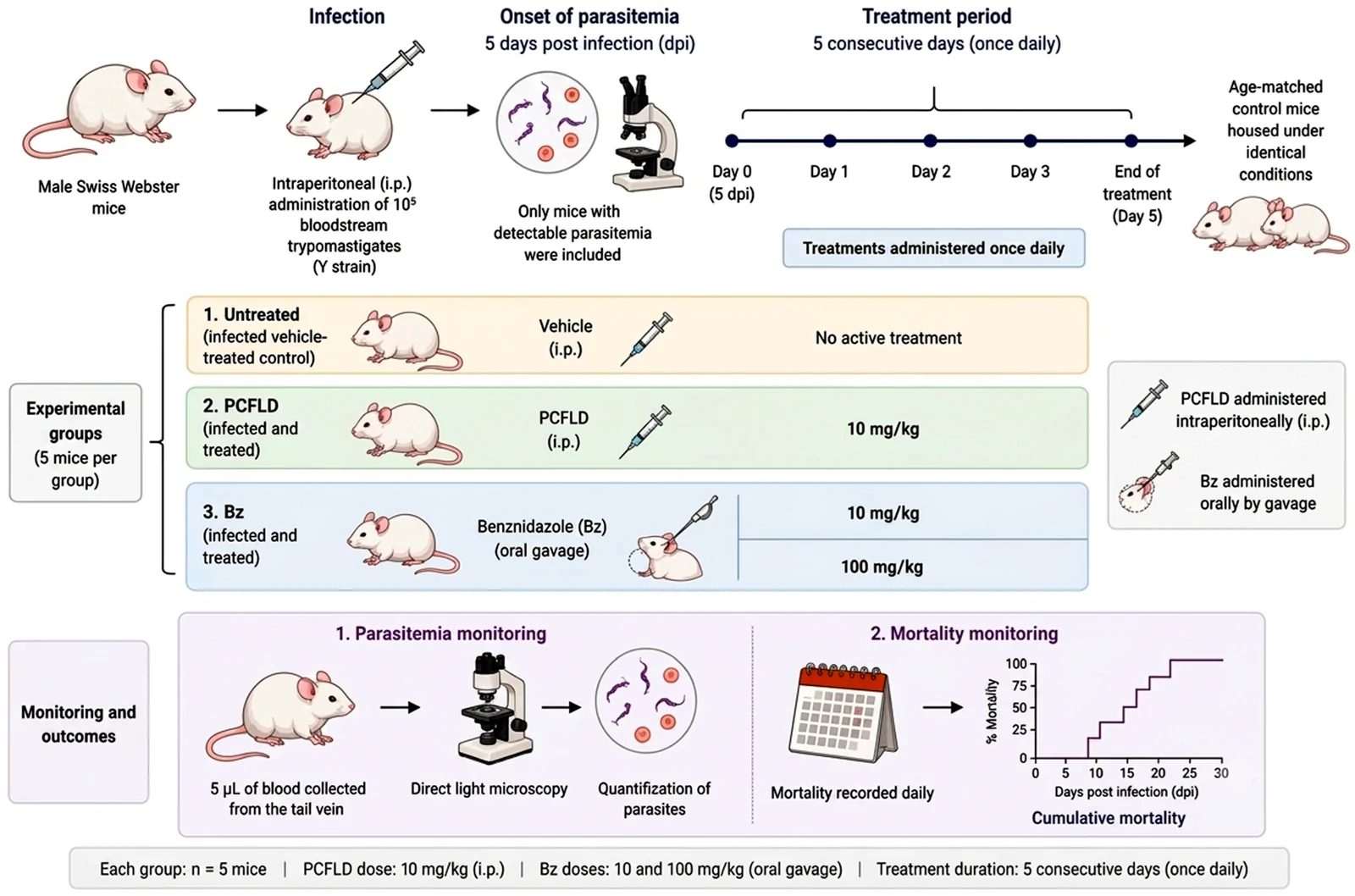
Experimental design for the in vivo evaluation of PCFLD against acute *T. cruzi* infection. Male Swiss Webster mice were infected intraperitoneally with 10^5^ bloodstream trypomastigotes (Y strain). Treatment began at 5 days post-infection (dpi), when parasitemia was first detected. Mice (*n* = 5/group) received vehicle, PCFLD (10 mg/kg, i.p.), or Bz (10 or 100 mg/kg, oral gavage) once daily for five consecutive days. Parasitemia was monitored by direct microscopy of 5 μL tail-vein blood, and cumulative mortality was recorded daily.

The doses of 10 and 100 mg/kg/day of Bz represent suboptimal and optimal doses of this reference drug, respectively, for in vivo mouse models of *T. cruzi* experimental infection [29]. Parasitemia was individually monitored by direct light microscopy through the quantification of parasites in 5 µL of blood collected from the tail vein. Mortality was recorded daily and expressed as cumulative mortality (% CM) (Figure 2) [55].

### 4.9. Analysis

The data analysis was performed using nonlinear regression analysis in GraphPad Prism software version 9.0 (GraphPad Software, San Diego, CA, USA). Concentration–response curves were generated, and the EC_50_ and LC_50_ values were calculated when applicable. Statistical comparisons between experimental groups were performed using one-way analysis of variance (ANOVA), and differences were considered statistically significant when *p* ≤ 0.05.

### 4.10. Ethics

All studies using animals were carried out in strict compliance with the guidelines established by the FIOCRUZ Committee of Ethics for the Use of Animals (CEUA/IOC-019/2023 approved on 9 November 2023) and were approved by CTNBio and CIBIO/IOC/FIOCRUZ for a Biosafety Quality Certificate (CQB 105/99) for the use of GMOs (*T. cruzi* Tulahuen strain transfected with β-galactosidase gene), and SisGen (AB5D582).

### 4.11. Artificial Intelligence

The authors used ChatGPT (GPT-5.6 Luna, OpenAI, San Francisco, CA, USA) to assist with the revision of grammar, language editing, and translation of selected sections of the manuscript. All scientific content, interpretation of results, and final text revisions were reviewed and approved by the authors, who take full responsibility for the content of this publication.

## 5. Conclusions

In conclusion, the findings obtained using the L929 and H9c2(2-1) cell models demonstrate that most of the evaluated natural products exhibit a favorable safety profile toward mammalian cells under the experimental conditions employed. The low cytotoxicity observed for most of the extracts, combined with antiparasitic activity against different forms of *T. cruzi*, highlights the potential as sources of promising antiparasitic candidates. Among the evaluated samples, the extract obtained from the inflorescences of *P. claussenianum* stood out due to its remarkable activity against both the intracellular amastigote and bloodstream trypomastigote forms of *T. cruzi*, associated with limited cytotoxic effects and a promising selectivity profile. Based on established criteria for antiparasitic drug discovery, these characteristics support the classification of the *P. claussenianum* inflorescence extract as a potential hit candidate, justifying its further evaluation in an in vivo model. In vivo assays further demonstrated its ability to reduce parasitemia in infected mice, reinforcing its potential for continued development. Further studies involving chemical characterization, isolation and identification of active constituents; evaluation of structure–activity relationships; and additional pharmacological investigations will be essential to elucidating its therapeutic potential and contribute to the development of new strategies for Chagas disease treatment.

## Figures and Tables

**Table 1:** Cytotoxicity profile of the reference drug and botanical extracts against L929 fibroblasts after 96 h of exposure. Extract codes consist of the plant species initials, followed by the plant part (Portuguese abbreviation) and extraction solvent (e.g., DBCM = *Dasyphyllum brasiliensis* stem extracted with methanol). A complete description of the extract codes is provided in Materials and Methods section.

Plant Species	Code	LC_50_ (µg/mL—Mean ± SD)
—	Bz	>120
*Schinus terebinthifolius* Raddi	STFE70	>120
	STFE70PAc	79.74 ± 23.27
*Dasyphyllum brasiliense* Spreng	DBFE	>120
	DBGE	>120
	DBCM	>120
	DBCH	>120
*ErecHitites valerianifolius* (Link ex Spreng.) DC	EVFE70	>120
*Licania tomentosa* Benth	LTSE	>120
	LTPFr	>120
*Garcinia brasiliensis* Mart	GBFE	>120
	GBCE	>120
*Erythrina speciosa* Andrew	ESCSE70	>120
*Laurus nobilis* L.	LNFE70	>120
	LNGE70	>120
*Nectandra oppositifolia* Nees	NOFE	>120
	NOGE	>120
	NOCs70	>120
*Couroupita guianensis* Aubl.	CouGFLE	>120
*Linum usitatissimum* L.	LUSE	>120
	LUSEpp	>120
	LUSEsb	>120
*Schwartzia brasiliensis* (Choisy) Bedell ex Gir.-Cañas	SBFE	>120
	SBFEPAc	>120
*Eugenia uniflora* L.	EUFE	>120
*Piper anisum* (*Otonia anisium*) (Spreng.) Angely	OAFE	>120
*Piper claussenianum* (Miq.) C. DC.	PCFH	>120
	PCFLD	72.18 ± 4.76
*Piper rivinoides* Kunth	PRFE	>120
*Piper umbellatum* L.	PUFE70	>120
	PUCE70	>120
*Manilkara zapota* (L.) P.Royen	MZFE70	>120
*Solanum paniculata* L.	SPFE70	>120
	SPGE70	>120
	SPFrE70	>120

**Table 2:** In vitro antiparasitic activity of natural product extracts and Bz against intracellular forms of *T. cruzi* (Tulahuen strain—DTU VI) at a fixed concentration of 20 μg/mL after 96 h of incubation.

Plant Species	Code	% Parasitism Decline (Mean ± DP)
—	Bz	89.29 ± 4.71
*Schinus terebinthifolius* Raddi	STFE70	5.65 ± 5.36
	STFE70PAc	6.51 ± 0.69
*Dasyphyllum brasiliense* Spreng	DBFE	7.69 ± 3.78
	DBGE	22.27 ± 8.64
	DBCM	7.75 ± 0.25
	DBCH	6.67 ± 0.61
*ErecHitites valerianifolius* (Link ex Spreng.) DC	EVFE70	7.02 ± 0.61
*Licania tomentosa* Benth	LTSE	8.67 ± 4.19
	LTPFr	4.80 ± 1.69
*Garcinia brasiliensis* Mart	GBFE	5.58 ± 2.50
	GBCE	9.18 ± 1.83
*Erythrina speciosa* Andrew	ESCSE70	6.42 ± 0.7
*Laurus nobilis* L.	LNFE70	10.73 ± 1.08
	LNGE70	8.24 ± 1.70
*Nectandra oppositifolia* Nees	NOFE	4.96 ± 1.79
	NOGE	4.77 ± 1.96
	NOCs70	7.69 ± 3.49
*Couroupita guianensis* Aubl.	CouGFLE	17.55 ± 5.70
*Linum usitatissimum* L.	LUSE	19.13 ± 0.38
	LUSEpp	5.23 ± 3.74
	LUSEsb	6.16 ± 2.85
*Schwartzia brasiliensis* (Choisy) Bedell ex Gir.-Cañas	SBFE	3.54 ± 1.86
	SBFEPAc	8.97 ± 0.84
*Eugenia uniflora* L.	EUFE	12.62 ± 4.76
*Piper anisum* (*Otonia anisium*) (Spreng.) Angely	OAFE	12.26 ± 5.30
*Piper claussenianum* (Miq.) C. DC.	PCFH	5.62± 3.06
	PCFLD	94.66 ± 9.23
*Piper rivinoides* Kunth	PRFE	1.54 ± 0.80
*Piper umbellatum* L.	PUFE70	2.06 ± 0.93
	PUCE70	10.37 ± 2.97
*Manilkara zapota* (L.) P.Royen	MZFE70	6.15 ± 1.30
*Solanum paniculata* L.	SPFE70	0.90 ± 0.89
	SPGE70	7.67 ± 0.1
	SPFrE70	9.60 ± 0.90

**Table 3:** Phenotypic activity (EC_50_ μg/mL) of *P. claussenianum* and Bz against intracellular forms (Tulahuen-β gal strain) and bloodstream trypomastigotes (BT, Y strain DTU II) of *T. cruzi* and cytotoxicity upon L929 cell line cultures and cardiac cells H9c2(2-1) (LC_50_, μg/mL), with their respective Selectivity Index (SI).

Sample Code	Intracellular Forms EC_50_ (µg/mL—Mean ± SD)	L929—LC_50_ (µg/mL—Mean ± SD)	SI	BT 24 h—EC_50_ (µg/mL—Mean ± SD)	H9c2—LC_50_ (µg/mL—Mean ± SD)	SI
Bz	0.17 ± 0.007	>120	>700	3.89 ± 1.19	ND	-
PCFLD	3.60 ± 0.36	72.18 ± 4.76	20	11.56 ± 5.88	>120	>11

ND: not determined.

**Table 4:** Botanical families and plant species investigated in this study, together with the corresponding extract codes, plant parts used for extraction, and solvents employed in the preparation of the extracts.

Family	Plant Species	Code *	Plant Part	Solvent
Anacardiaceae	*Schinus terebinthifolius* Raddi	STFE70	Leaf	70% Ethanol
		STFE70PAc	Leaf	70% Ethanol—Ethyl Acetate
Asteraceae	*Dasyphyllum brasiliense* Spreng	DBFE	Leaf	70% Ethanol
		DBGE	Branch	70% Ethanol
		DBCM	Stem	99% Methanol
		DBCH	Stem	95% Hexane
	*ErecHitites valerianifolius* (Link ex Spreng.) DC	EVFE70	Leaf	70% Ethanol
Chrysobalanaceae	*Licania tomentosa* Benth	LTSE	Seed	70% Ethanol
		LTPFr	Fruit	70% Ethanol
Clusiaceae	*Garcinia brasiliensis* Mart	GBFE	Leaf	70% Ethanol
		GBCE	Stem	70% Ethanol
Fabaceae	*Erythrina speciosa* Andrew	ESCSE70	Bark	70% Ethanol
Lauraceae	*Laurus nobilis* L.	LNFE70	Leaf	70% Ethanol
		LNGE70	Branch	70% Ethanol
	*Nectandra oppositifolia* Nees	NOFE	Leaf	70% Ethanol
		NOGE	Branch	70% Ethanol
		NOCs70	Bark	70% Ethanol
Lecythidaceae	*Couroupita guianensis* Aubl.	CouGFLE	Branch	70% Ethanol
Linaceae	*Linum usitatissimum* L.	LUSE	Seed	70% Ethanol
		LUSEpp	Seed	70% Ethanol
		LUSEsb	Seed	70% Ethanol
Marcgraviaceae	*Schwartzia brasiliensis* (Choisy) Bedell ex Gir.-Cañas	SBFE	Leaf	70% Ethanol
		SBFEPAc	Leaf	70% Ethanol—Ethyl Acetate
Myrtaceae	*Eugenia uniflora* L.	EUFE	Leaf	70% Ethanol
Piperaceae	*Piper anisum* (*Otonia anisium*) (Spreng.) Angely	OAFE	Leaf	70% Ethanol
	*Piper claussenianum* (Miq.) C. DC.	PCFH	Leaf	95% Hexane
		PCFLD	Inflorescences	>99.8% Dichloromethane
	*Piper rivinoides* Kunth	PRFE	Leaf	70% Ethanol
	*Piper umbellatum* L.	PUFE70	Leaf	70% Ethanol
		PUCE70	Bark	70% Ethanol
Sapotaceae	*Manilkara zapota* (L.) P.Royen	MZFE70	Leaf	70% Ethanol
Solanaceae	*Solanum paniculata* L.	SPFE70	Leaf	70% Ethanol
		SPGE70	Branch	70% Ethanol
		SPFrE70	Fruit	70% Ethanol

* The extract codes were generated using the initials of the plant species name, followed by an abbreviation of the plant part (in Portuguese) and the extraction solvent. For example, DBCM corresponds to *Dasyphyllum brasiliensis* stem (caule in Portuguese) extracted with methanol.

## Data Availability

The original contributions presented in this study are included in the article/Supplementary Material. Further inquiries can be directed to the corresponding author(s).

## Supplementary Materials

The following supporting information can be downloaded at: https://www.mdpi.com/article/10.3390/molecules31162790/s1, Figure S1. HPLC chromatographic profile (chemical fingerprint) of the PCFLD (*Piper claussenianum* (Miq.) C. DC.) extract. RT 42.2 min (5,7-dihydroxyflavanone); RT 43.5 min (2′,6′-dihydroxy-4′-methoxychalcone); RT 44.7 min (7-hydroxy–5–methoxyflavanone). Figure S2. HPLC chromatographic profile (chemical fingerprint) of compounds isolated from the dichloromethane extract of *P. claussenianum* the PCFLD (*Piper claussenianum* (Miq.) C. DC.) extract. The 1H NMR spectrum of 5,7-dihydroxyflavanone. (**A**); 1H NMR spectrum of 2′,6′-dihydroxy-4′-methoxychalcone (**B**); 1H NMR spectrum of 7-hydroxy–5–methoxyflavanone (**C**). Table S1. Collection sites, geographic coordinates, and voucher specimen numbers of the plant species evaluated in this study.

## Abbreviations

The following abbreviations are used in this manuscript:

NTD	Neglected Tropical Disease
CD	Chagas disease
*T. cruzi*	*Trypanosoma cruzi*
CCC	Chronic Chagas cardiomyopathy
Bz	Benznidazole
Nf	Nifurtimox
SI	Selectivity Index
DMSO	Dimethyl sulfoxide
DMEM	Dulbecco’s Modified Eagle Medium
PBS	phosphate-buffered saline
min	minute
CPRG	chlorophenol red-β-D-galactopyranoside
h	Hour
°C	Celsius degrees
ICTB	Institute of Science and Technology in Biomodels
dpi	Dys post infection
