# Supplementary material for: Phenotypic Analysis of the Anti-T. cruzi Activity of Natural Products Obtained from Brazilian Botanical Sources
Source: Molecules. 2026 Aug 11;31(16):2790. doi: 10.3390/molecules31162790 (PMC13515798; doi:10.3390/molecules31162790)
Supplement: Supplementary file 1 [file molecules-31-02790-s001.zip › molecules-4443142-supplementary.pdf]

Supplementary Material

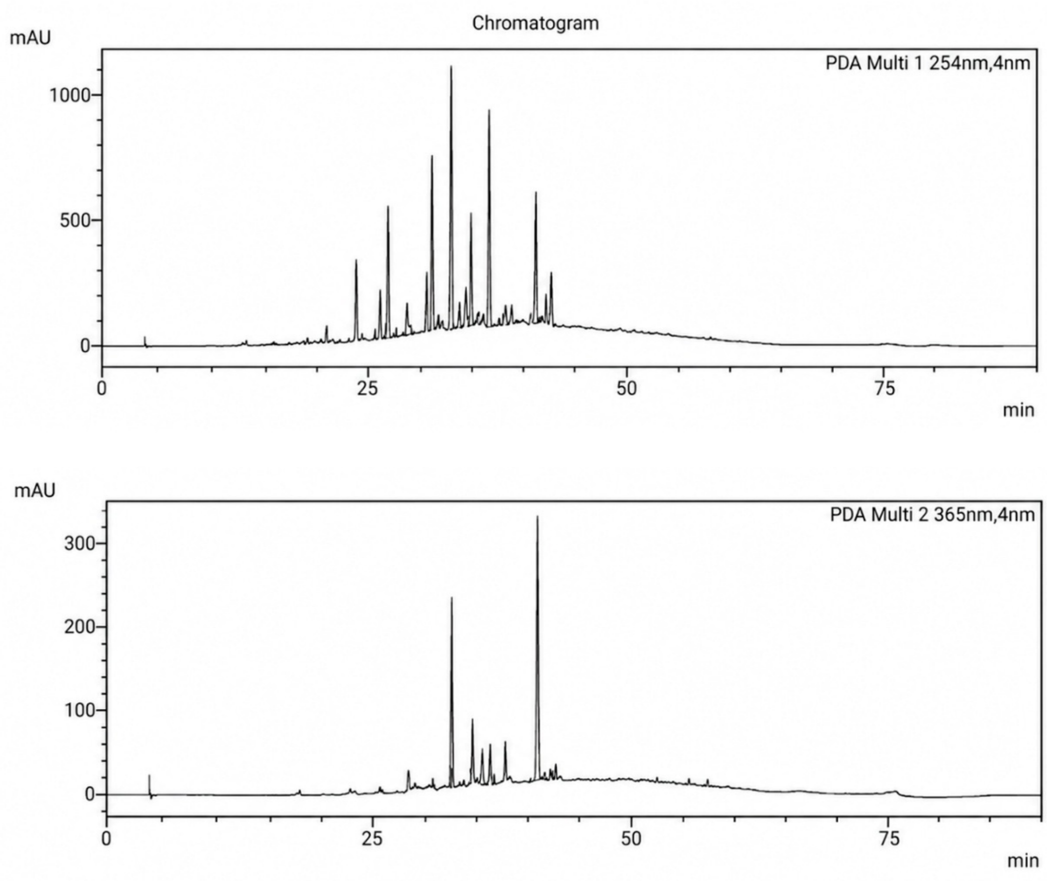

Figure S1. HPLC chromatographic profile (chemical fingerprint) of the PCFLD (*Piper claussenianum (Miq.) C. DC.*) extract. RT 42,2min (5,7-dihydroxyflavanone); RT 43,5min (2′,6′ - dihydroxy - 4′- methoxychalcone); RT 44,7min (7-hydroxy – 5 – methoxyflavanone).

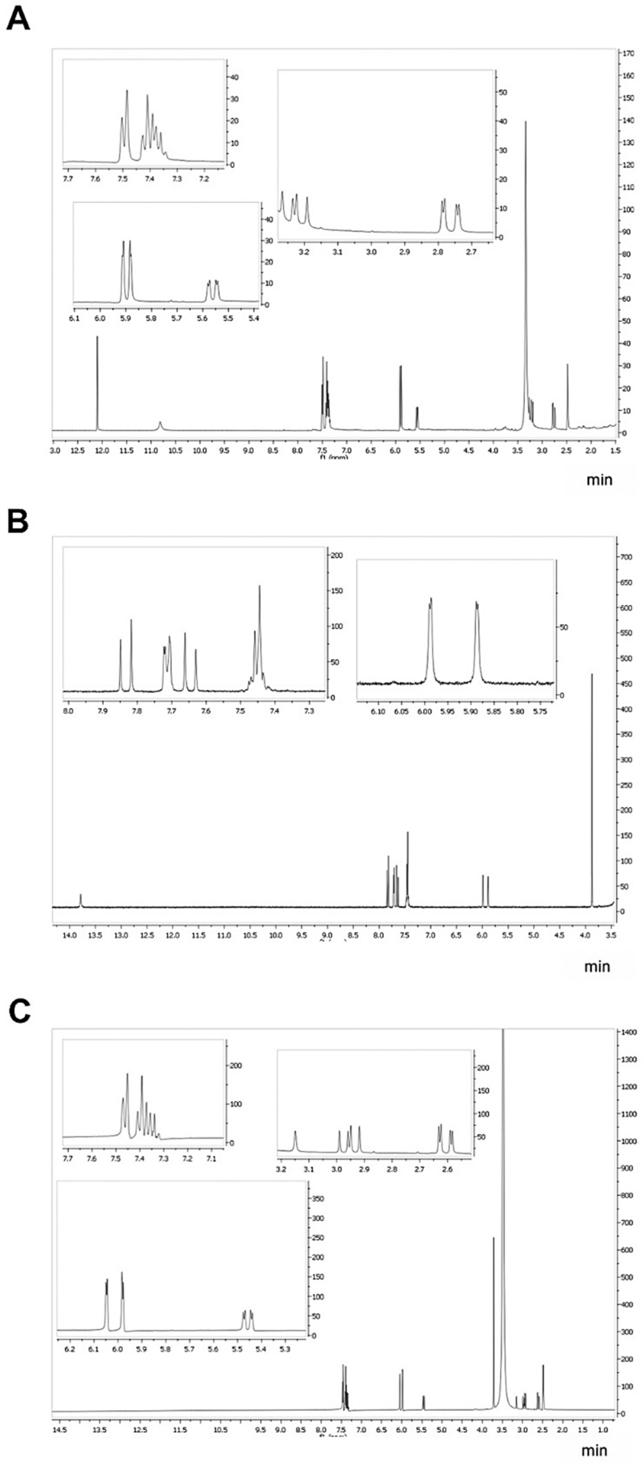

Figure S2. HPLC chromatographic profile (chemical fingerprint) of compounds isolated from the dichloromethane extract of *P. claussenianum* the PCFLD (*Piper claussenianum* (Miq.) C. DC.) extract. ^{1}H NMR spectrum of 5,7-dihydroxyflavanone. (**A**); ^{1}H NMR spectrum of 2′,6′ - dihydroxy - 4′- methoxychalcone (**B**); ^{1}H NMR spectrum of 7-hydroxy – 5 – methoxyflavanone (**C**).

Table S1 . Collection sites, geographic coordinates, and voucher specimen numbers of the plant species evaluated in this study.

Plant species	Collection site	Geographic coordinates	Voucher specimen number
Couroupita guianensis Aubl.	Fiocruz, Rio de Janeiro (RJ)	22°52'24.7"S, 43°14'49.0"W	RB 136147
Dasyphyllum brasiliense Spreng	Matipó, Minas Gerais (MG)	20°21'16.2"S, 42°18'58.3"W	RB 632599
Erechitites valerianifolius Link ex Spreng.) DC	Fiocruz, Petrópolis (RJ)	22°30'54.2"S, 43°11'13.7"W	CBPM 1790
*Erythrina speciosa*Andrew	Fiocruz, Petrópolis (RJ)	22°30'54.2"S, 43°11'13.7"W	RFA 47937
Eugenia uniflora L.	Fiocruz, Rio de Janeiro (RJ)	22°52'38.0"S, 43°14'56.1"W	RFA 39909
Garcinia brasiliensis Mart.	Matipó, Minas Gerais (MG)	20°21'16.2"S, 42°18'58.3"W	RB 304853
Laurus nobilis L.	Matipó, Minas Gerais (MG)	20°21'16.2"S, 42°18'58.3"W	RB 249647
Licania tomentosa Benth	Fiocruz, Rio de Janeiro (RJ)	22°52'33.8"S, 43°14'52.7"W	RBcarpo 2573
Linum usitatissimum L.	Fiocruz, Rio de Janeiro (RJ)	22°52'33.8"S, 43°14'52.7"W	RB 621974
Manilkara zapota (L.) P.Royen	Fiocruz, Rio de Janeiro (RJ)	22°52'37.8"S, 43°14'58.9"W	CBPM 1711
Nectandra oppositifolia Nees	Fiocruz, Petrópolis (RJ)	22°30'54.2"S, 43°11'13.7"W	RFA 46395
Piper anisum (*Otonia anisium*) (Spreng.) Angely	Matipó, Minas Gerais (MG)	20°21'16.2"S, 42°18'58.3"W	RB 393494
Piper claussenianum (Miq.)C. DC.	Matipó, Minas Gerais (MG)	20°21'16.2"S, 42°18'58.3"W	RB 606975
Piper rivinoides Kunth	Colônia Juliano Moreira, Rio de Janeiro (RJ)	22°56'23.8"S, 43°24'16.2"W	RB 554171
Piper umbellatum L.	Fiocruz, Rio de Janeiro (RJ)	22°52'33.8"S, 43°14'52.7"W	CBPM 1710
Schinus terebinthifolius Raddi	Colônia Juliano Moreira, Rio de Janeiro (RJ)	22°56'23.8"S, 43°24'16.2"W	RB 444626
Schwartzia brasiliensis (Choisy) Bedell ex Gir.-Cañas.	Fiocruz, Rio de Janeiro (RJ)	22°52'48.4"S, 43°14'25.4"W	R 218186
Solanum paniculata L.	Matipó, Minas Gerais (MG)	20°21'16.2"S, 42°18'58.3"W	RBR35274
